# The Impact of Recreational Cannabis Legalization on Cannabis Use and Associated Outcomes: A Systematic Review

**DOI:** 10.1177/11782218231172054

**Published:** 2023-05-09

**Authors:** Kyra N Farrelly, Jeffrey D Wardell, Emma Marsden, Molly L Scarfe, Peter Najdzionek, Jasmine Turna, James MacKillop

**Affiliations:** 1 Department of Psychology, York University, Toronto, ON, Canada; 2 Peter Boris Centre for Addictions Research, St. Joseph’s Healthcare Hamilton, McMaster University, Hamilton, ON, Canada; 3 Institute for Mental Health Policy Research, Centre for Addiction and Mental Health, Toronto, ON, Canada; 4 Department of Psychiatry, University of Toronto, Toronto, ON, Canada; 5 Michael G. DeGroote Centre for Medicinal Cannabis Research, McMaster University & St. Joseph’s Healthcare Hamilton, Hamilton, ON, Canada; 6 Homewood Research Institute, Guelph, ON, Canada

**Keywords:** Cannabis, marijuana, legalization, attitudes, health, crime, driving

## Abstract

**Background:**

Recreational cannabis legalization has become more prevalent over the past decade, increasing the need to understand its impact on downstream health-related outcomes. Although prior reviews have broadly summarized research on cannabis liberalization policies (including decriminalization and medical legalization), directed efforts are needed to synthesize the more recent research that focuses on recreational cannabis legalization specifically. Thus, the current review summarizes existing studies using longitudinal designs to evaluate impacts of recreational cannabis legalization on cannabis use and related outcomes.

**Method:**

A comprehensive bibliographic search strategy revealed 61 studies published from 2016 to 2022 that met criteria for inclusion. The studies were predominantly from the United States (66.2%) and primarily utilized self-report data (for cannabis use and attitudes) or administrative data (for health-related, driving, and crime outcomes).

**Results:**

Five main categories of outcomes were identified through the review: cannabis and other substance use, attitudes toward cannabis, health-care utilization, driving-related outcomes, and crime-related outcomes. The extant literature revealed mixed findings, including some evidence of negative consequences of legalization (such as increased young adult use, cannabis-related healthcare visits, and impaired driving) and some evidence for minimal impacts (such as little change in adolescent cannabis use rates, substance use rates, and mixed evidence for changes in cannabis-related attitudes).

**Conclusions:**

Overall, the existing literature reveals a number of negative consequences of legalization, although the findings are mixed and generally do not suggest large magnitude short-term impacts. The review highlights the need for more systematic investigation, particularly across a greater diversity of geographic regions.

## Introduction

Cannabis is one of the most widely used substances globally, with nearly 2.5% of the world population reporting past year cannabis use.^1^ Cannabis use rates are particularly high in North America. In the U.S., 45% of individuals reported ever using cannabis and 18% reported using at least once annually in 2019.^2,3^ In Canada, approximately 21% of people reported cannabis use in the past year use in 2019.^4^ In terms of cannabis use disorder (CUD), a psychiatric disorder defined by clinically significant impairment in daily life due to cannabis use,^5^ ~5.1% of the U.S. population ages 12+ years met criteria in 2020, with ~13.5% of individuals ages 18 to 25 years meeting criteria.^6^

Overall, rates of cannabis use have shown long-term increasing trends among several age groups in North America.^7-9^ Moreover, research has revealed recent cannabis use increases in at risk populations, such as individuals with depression and pregnant women.^10,11^ Parallel to increased cannabis use over time, rates of cannabis-related consequences have also increased across Canada and the U.S., including cannabis dependence and CUD,^8,12^ crime rates (eg, increased possession charges),^8^ and cannabis-impaired driving (and, lower perception of impairment and risk from cannabis use).^11,13,14^ Further, cannabis use poses a risk for early-onset or use during adolescence as there is evidence that cannabis use in adolescence is linked with poorer cognitive performance, psychotic disorders, and increased risk of mood and addictive disorders.^15^ With the rates of negative consequences from cannabis use increasing, particularly in North America where cannabis has become legal in many parts of the US and all of Canada, understanding the role of cannabis legalization in these changes is crucial to inform ongoing changes in cannabis policies worldwide.

The legal status of cannabis varies widely across countries and regions. Although cannabis is largely illegal at the global level, policies surrounding cannabis use are becoming steadily liberalized. Decriminalization (reduced penalties for self-use but not distribution) is more widespread worldwide, including in the Netherlands, Portugal, and parts of Australia. Medical legalization is also seen in Peru, Germany, New Zealand, the Netherlands and across many U.S. states. To date, Canada, Uruguay, and Malta are the only 3 countries to legalize recreational cannabis use at the national level. Further, individual U.S. states began legalizing recreational cannabis in 2012, with nearly half of U.S. states having legalized recreational cannabis by 2023. As national and subnational recreational legalization continues to gain support and take effect, understanding the consequences of such major regulatory changes is crucial to informing ongoing policy changes.

There are arguments both for and against recreational cannabis legalization (RCL). Common pro-legalization arguments involve increasing regulatory control over product distribution, weakening organized crime, reducing burden and inequality in the criminal justice system, and generating economic benefits such as tax revenues and commercial activity.^16^ Furthermore, as cannabis obtained from illicit markets is of varying and unknown potency,^17^ cannabis legalization may help better regulate the potency and quality of cannabis products.^18^ On the other hand, there are anti-legalization arguments such as the possibility of legalization leading to increased use among youth and increased cannabis-impaired driving.^16^ A nationally representative survey in the U.S. found that pro-legalization arguments were perceived to be more persuasive than public health anti-legalization arguments in a U.S. nationally representative survey,^19^ suggesting policymaker concerns regarding RCL do not seem to hold as much weight in the general public. However, while research may be increasing surrounding the impacts of RCL, the general consensus of if RCL leads to more positive or negative consequences is unclear.

With RCL becoming more prevalent globally, the impacts it may have on a variety of health-related outcomes are of critical importance. Prevalence of cannabis use is of course a relevant issue, with many concerned that RCL will cause significant spikes in rates of cannabis use for a variety of groups, including youth. However, current studies have revealed mixed evidence in the U.S.,^20,21^ thus there is a need to synthesize the extant literature to better understand the balance of evidence and potential impacts of RCL across different samples and more diverse geographic areas. Another common question about RCL is whether it will result in changes in attitudes toward cannabis. These changes are of interest as they might forecast changes in consumption or adverse consequences. Similarly, there are concerns surrounding RCL and potential spill-over effects that may influence rates of alcohol and other substance use.^22^ Thus, there remains a need to examine any changes in use of other substance use when studying effects of RCL.

Beyond changes in cannabis and other substance use and attitudes, health-related impacts of RCL are important to consider as there are links between cannabis use and adverse physical and mental health consequences (eg, respiratory and cardiovascular diseases, psychosis).^23^ Additionally, emergency service utilization associated with cannabis consumption is a frequent concern associated with RCL, particularly due to the spikes in admissions following RCL in Colorado.^24^ However, the rates of cannabis-related emergency service admissions more globally (eg, in legal countries like Canada and Uruguay) have not been fully integrated into summaries of the current literature. Finally, another health-related consequence of RCL is potential impacts on opioid use. While opioid-related outcomes can fall into substance use, they are considered health-related for this review as much of the discussion surrounding RCL and opioids involve cannabis substituting opioid use for medicinal reasons or using cannabis as an alternate to prescription opioids in the healthcare system. The current opioid crisis is a global public health problem with serious consequences. While there is evidence that medicinal cannabis may reduce prescription opioid use^25^ and that cannabis may be a substitute for opioid use,^26^ the role of recreational cannabis legalization should also be examined as the 2 forms of cannabis use are not interchangable^27^ and have shown unique associations with prescription drug use.^28^ Thus, there is a need to better understand how and if RCL has protective or negative consequences on opioid-related outcomes.

Due to the impairing effects of cannabis on driving abilities and the relationship with motor vehicle accidents,^29^ another important question surrounding RCL is how these policy changes could result in adverse driving-related outcomes. An understanding of how RCL could influence impaired driving prevalence is needed to give insight into how much emphasis jurisdictions should put on impaired driving rates when considering RCL implementation. A final consequence of RCL that is often debated but requires a deeper understanding is how it impacts cannabis-related arrest rates. Cannabis-related arrests currently pose a significant burden on the U.S. and Canadian justice system.^30,31^ Theoretically, RCL may ease the strain seen on the justice system and have positive trickle-down effects on criminal-related infrastructure. However, the overall implications of RCL on arrest rates is not well understood and requires a systematic evaluation. With the large number of RCL associated outcomes there remains a need to synthesize the current evidence surrounding how RCL can impact cannabis use and other relevant outcomes

### Present review

Currently, no reviews have systematically evaluated how RCL is associated with cannabis-use changes across a variety of age groups as well as implications on other person- or health-related outcomes. The present review aims to fill an important gap in the literature by summarizing the burgeoning research examining a broad range of consequences of RCL across the various jurisdictions that have implemented RCL to date. Although previous reviews have considered the implications of RCL,^32,33^ there has recently been a dramatic increase in studies in response to more recent changes in recreational cannabis use policies, requiring additional efforts to synthesize the latest research. Further, many reviews focus on specific outcomes (eg, parenting,^34^ adolescent use^35^). There remains a need to systematically summarize how RCL has impacted a variety of health-related outcomes to develop a more comprehensive understanding of the more negative and positive outcomes of RCL. While a few reviews have examined a broad range of outcomes such as cannabis use, related problems, and public health implications,^32,33^ some reviews have been limited to studies from a single country or published in a narrow time window.^32^ Thus, a broader review is necessary to examine multiple types of outcomes from studies in various geographic regions. Additionally, a substantial amount of the current literature examining the impact of RCL relies on cross-sectional designs (eg, comparing across jurisdictions with vs without recreational legalization) which severely limit any conclusions about causal associations. Thus, given its breadth, the current systematic review is more methodologically selective by including only studies with more rigorous designs (such as longitudinal cohort studies), which provide stronger evidence regarding the effects of RCL. In sum, the aim of the current review was to characterize the health-related impacts of RCL, including changes in these outcomes in either a positive or negative direction.

## Method

The review is compliant with the Preferred Reporting Items for Systematic Reviews and Meta-Analyses (PRISMA^36^). Full-text extraction was initiated immediately following article search, therefore the protocol was not registered with PROSPERO. Relevant articles on cannabis legalization were principally identiﬁed using the Boolean search terms (“cannabis” OR “marijuana” OR “THC” OR “marihuana”) AND “legalization” AND (“recreational” OR “non-medical” OR “nonmedical”) AND (“longitudinal” OR “pre-post” OR “prospective” OR “timeseries” OR “cohort”). The search was conducted using PubMed/MEDLINE, EMBASE, and PsycINFO through November 2022. Relevant studies identified through secondary means (eg, prior knowledge of a relevant publication, articles brought to the authors’ attention) were also included for screening. Titles and abstracts resulting from the initial search were screened in Covidence (Veritas Health Innovation Inc) by 2 reviewers for suitability for full-text review and ﬁnal inclusion. Conflicts were discussed by both reviewers and a final decision was made by consensus. Following screening, reviewers read and extracted relevant data. To be included, an article was required to meet the following criteria: (i) an original empirical research article published in a peer-reviewed journal; (ii) written in (or available in) English; (iii) RCL serves as an independent variable; (iv) quantitative study design that clearly permitted the evaluation of the role of RCL with a more rigorous non-cross-sectional study design (eg, pre- vs post-legalization, longitudinal, cohort, interrupted time series, etc.); and (v) reports on health-related outcomes (ie, changes in consumption or attitudes, as opposed to changes in price or potency).

RCL related outcomes that were considered were those specifically involving the behavior, perceptions, and health of individuals. Population-level outcomes (eg, health-care utilization or impaired driving) were considered eligible for inclusion as they involve the impacts that legalization has on individual behavior. Thus, economic- or product-level outcomes that do not involve individual behavior (eg, cannabis prices over time, changes in cannabis strain potency) were considered out of scope. The outcome groups were not decided ahead of time and instead 5 main themes in outcomes emerged from our search and were organized into categories for ease of presentation due to the large number of studies included.

Studies that examined medicinal cannabis legalization or decriminalization without recreational legalization, and studies using exclusively a cross-sectional design were excluded as they were outside the scope of the current review. The study also excluded articles that classified RCL as the passing of legal sales rather than implementation of RCL itself as RCL is often distinct from introduction of legal sales, or commercialization. Thus, we excluded studies examining commercialization as they were outside the scope of the current review.

## Results

### Characteristics of the literature

**Figure 1.**
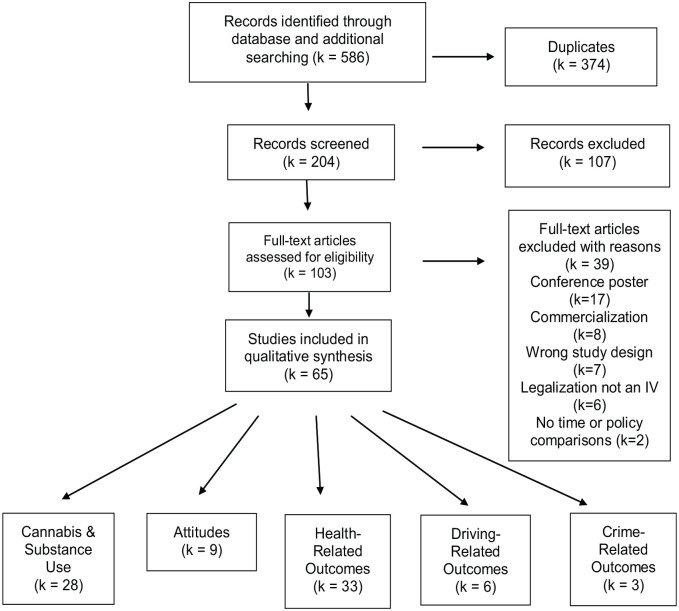
Preferred Reporting Items for Systematic Review and Meta-Analyses (PRISMA) study flow diagram.

The search revealed 65 relevant articles examining RCL and related outcomes (see Figure 1). There were 5 main themes established: cannabis use and other substance use behaviors (*k* = 28), attitudes toward cannabis (*k* = 9), health-related outcomes (*k* = 33), driving related impacts (*k* = 6), and crime-related outcomes (*k* = 3). Studies with overlapping themes were included in all appropriate sections. Most studies (66.2%) involved a U.S. sample, 32.3% examined outcomes in Canada, and 1.5% came from Uruguay. Regarding study design, the majority (46.2%) utilized archival administrative data (ie, hospital/health information across multiple time points in one jurisdiction) followed by cohort studies (18.5%). The use of administrative data was primarily used in studies examining health-related outcomes, such as emergency department utilization. Studies examining cannabis use or attitudes over time predominantly used survey data. Finally, both driving and crime related outcome studies primarily reported findings with administrative data.

### Changes in cannabis and other substance use

Cannabis and other substance use changes represented the second largest number of studies, with 28 articles identified. Studies examining changes in cannabis use behaviors were divided by subpopulation (ie, adolescents, young adults, general population adults, clinical populations, and maternal use; see Table 1). Finally, we separately summarized studies reporting changes in concurrent use of other substances, and routes of cannabis administration.

**Table 1.** Studies investigating the role of recreational cannabis legalization on cannabis and other substance consumption.

Author	Year	Location	Date of legalization	Study design	Sample	Brief findings
*Cannabis use in adolescents*						
Duan et al	2022	U.S.		Longitudinal	N = 19 503	In states with RCL adolescents who never used cannabis but used e-cigarettes were more likely to use cannabis than those living in states without recreational cannabis legalization.
Estoup et al	2016	Washington	2012	Cohort	N = 262	Cannabis-related consequences significantly increased following RCL. There was not a significant effect of frequency of cannabis use.
Gunadi et al	2022	U.S.	2016	Longitudinal	N = 21 863	Significant association between RCL and transition from non-users to cannabis users when compared to states with no medical or recreational cannabis legalization and states with no legalization combined with those with medical cannabis legalization, but not when compared to states with medical cannabis legalization only.
Mason et al	2016	Washington	2012	Cohort	N = 238	RCL cohort had increased cannabis use at follow-up compared to pre-RCL, but this increase was not significant. There were positive, but not significant, cohort effects for cannabis use.
Paschall et al	2022	California	2016	Repeated cross-sectional	N = 3 319 329	Adolescent alcohol and cannabis co-users had a significant increase in the frequency of past 30-day cannabis use following RCL.
Rusby et al	2018	Oregon	2015	Cohort	N = 444	RCL cohort was more likely to increase their intent to use cannabis overtime, while the pre-RCL cohort was less likely to increase willingness and intent to use. RCL was not associated with initiating cannabis use. The RCL cohort did have significant increases in cannabis use compared to pre-RCL.
Stormshak et al	2019	U.S.		Cohort	N = 1438	Post-RCL cohort had higher odds of cannabis use compared to the pre-RCL cohort over time. Use decreased over time for pre-RCL but increased post-RCL. However, patterns of use were similar in cohorts.
Vignault et al	2021	Quebec	2018	Archival administrative data	N = 2615	No significant increase in the frequency of or prevalence of cannabis use following RCL.
Yu et al	2020	U.S.		Cohort	N = 749 152	RCL was not significantly associated with period effects for cannabis use, but medical legalization was.
Zuckermann et al	2021	Canada	2018	Repeated cross-sectional	N = 102 685	Adolescents had increased odds of ever using cannabis in the year following RCL in the cross-sectional data^28^. However, the longitudinal sample revealed no significant differences in the odds of ever use, current use, and regular use of cannabis post-RCL.
*Cannabis use in young adults*						
Bailey et al	2020	Washington	2012	Longitudinal	N = 281	RCL predicted a higher likelihood of past-year cannabis use.
Barker & Moreno	2021	Washington & Wisconsin	2012	Longitudinal cohort	N = 338	Significant association between RCL and increased cannabis use. The rate of students ever using cannabis did not change, however, in those who had used cannabis prior to RCL, the proportion of students using in the past 28-days increased faster following RCL in Washington (legal-state) when compared with the rate of increase in Wisconsin (non-legal state).
Han & Seo	2022	U.S.		Longitudinal cohort	N = 6155	In a sample of young adults who had never vaped cannabis at the time of recruitment results revealed that cannabis use in the past year did not differ in states with or without RCL, although, those living in states with RCL did show a larger increase in rates of cannabis vaping across time, compared to those in non-RCL states.
Kerr et al	2017	Oregon	2015	Repeated cross-sectional	N = 10 924	Rates of cannabis use significantly increased following RCL but use also increased over time in non-legal states. Oregon students with heavy alcohol use had greater increases in recent use. Among heavy drinker’s RCL had a greater impact on cannabis use for minors. No support that first year students experience a greater effect of RCL on use. RCL was not associated with changes in cigarette and alcohol use.
*Cannabis use in adults*						
Gali et al	2021	California	2016	Longitudinal cohort	N = 429	Past 30-day cannabis use increased significantly 1-month post-RCL and remained elevated 6-months post-RCL.
Gunadi et al	2022	U.S.	2016	Longitudinal cohort	N = 21, 863	In adults, there was an association between legalization and transition from non-users to cannabis users and non-users to weekly users when compared to states with no medical or recreational cannabis legalization and states with no legalization combined with those with medical cannabis legalization.
Kerr et al	2018	U.S.	2018	Repeated cross-sectional	N = 37 359	There was a non-significant increase in cannabis use post-RCL. Rates of simultaneous cannabis and alcohol use did not increase with RCL. Comparison studies found evidence of some increase in cannabis use 12 months after RCL. Past year cannabis use rates started increasing prior to RCL.
Turna et al	2021	Ontario	2018	Longitudinal	N = 1502	For non-users prior to RCL, there were significant increases in cannabis use frequency, quantity of cannabis used, and severity of cannabis misuse following RCL. The opposite pattern was seen for those reporting cannabis use prior to RCL, with significant decreases in frequency of use, quantity, and misuse.
Vignault et al	2021	Quebec	2018	Archival administrative data	N = 2615	No significant increase in the frequency of or prevalence of cannabis use following RCL.
*Cannabis use in clinical populations*						
Geoffrion et al	2021	British Columbia	2018	Archival administrative data	N = 3705	Cannabis use rates increased from pre- to post-RCL for women with pelvic pain.
Grigorian et al	2019	California	2016	Archival administrative data	N = 21 173	The rate of adult positive THC screens increased post-RCL. Pediatric positive THC screens were non-significant.
Hawke & Henderson	2021	Ontario	2018	Cohort	N = 269	In a sample of youth in an outpatient addictions treatment program, there was no change in the rate of cannabis use following RCL.
Hawley et al	2019	British Columbia	2018	Repeated cross-sectional	N = 1673	There was a significant increase in the prevalence of current cannabis use after RCL among cancer patients.
Pusateri et al	2022	Colorado & Washington	2012	Archival administrative data	N = 18 545	There was a significant increase of irritable bowel disease patients reporting cannabis use post-RCL.
Rosic et al	2021	Ontario	2018	Repeated cross-sectional	N = 1390	In individuals receiving treatment for opioid use disorder, cannabis use was compared for those recruited 6 months before or after RCL with no significant changes in the prevalence or frequency of self-reported or urine screen-detected cannabis use following RCL.
*Maternal cannabis use*						
Grant et al	2018	Washington	2012	Cohort	N = 1359	Increases in cannabis use in mothers who used substances during pregnancy at treatment exit post-RCL. Post-RCL cohort more likely to report cannabis use 30 days following exit compared to pre-RCL-cohort. Post-RCL cohort also less likely to quit cannabis use and more likely to have used from enrollment to exit. Post-RCL cohort who initiated use during treatment used about 3x more than Pre-RCL cohort.
Lee et al	2022	California	2016	Cohort	N = 466	Urine screen-detected cannabis use during pregnancy increased from 6% to 11% following RCL.
Yee et al	2021	U.S.		Cohort	N = 2926	No significant difference in cannabis or alcohol use associated with RCL in women living with HIV during pregnancy or the postpartum period.
*Other substance use*						
Bailey et al	2020	Washington	2012	Longitudinal	N = 281	RCL predicted a higher likelihood of alcohol use. RCL was not significantly associated with past-year cigarette use.
Grigorian et al	2019	California	2016	Archival administrative data	N = 21 173	There was no difference for alcohol and other drug screens in adults post-RCL. Post-RCL there was increased rate of benzodiazepine and barbiturate screens for pediatrics.
Hawke & Henderson	2021	Ontario	2018	Cohort	N = 269	No significant effect of RCL on rates of alcohol or illicit drug use.
Kerr et al	2017	Oregon	2015	Repeated cross-sectional	N = 10 924	Among heavy drinker’s RCL had a greater impact on cannabis use for minors. RCL was not associated with changes in cigarette and alcohol use.
Mason et al	2016	Washington	2012	Cohort	N = 238	The pre-RCL cohort had higher past month cigarette use at follow-up compared to the RCL cohort. Alcohol use was also greater for the pre-RCL cohort but not significantly. There were negative and significant cohort effects for alcohol and cigarette use.
Paschall et al	2022	California	2016	Repeated cross-sectional	N = 3 319 329	Among 7th, 9th, and 11th grade students in the U.S., RCL was associated with a 6% increase in the odds of past 30-day alcohol and cannabis co-use. The association was even stronger in students with past 30-day alcohol use and heavy drinking. However, among past 30-day cannabis users, RCL was associated with a 24% reduction in co-use.
*Route of administration*						
Gali et al	2021	California	2016	Longitudinal cohort	N = 429	Smoking, vaping, and edibles (in that order) were the most frequent modes of cannabis use pre- and post-RCL. The least common mode of cannabis use was blunts, which declined following RCL.
Zuckerman et al	2021	Canada	2018	Cohort	N = 2953	Changes in the number of different modes of cannabis use reported by high school students showed that 31.3% of students maintained a single mode of use, 14.3% maintained multiple modes of use, 42.3% expanded and 12.1% reduced their modes of use pre- and post-RCL.

Author, author of article; Year, publication year of article; Location, jurisdiction article data was collected in; Date of Legalization, year legalization was enacted in jurisdiction; Sample, total N of article sample; RCL, Recreational Cannabis Legalization.

#### Cannabis use changes in adolescents (~12-17)

Ten studies examined changes in cannabis use among adolescents and found that changes in the rates of use were inconsistent following RCL. Gunadi et al^37^ found an association between RCL and more pronounced transition from non-use to cannabis use when compared to states with no legalization and those with medical cannabis legalization (*P* ⩽ .001) combined, but not when compared to states with medical cannabis legalization only. Another study found that in states with RCL adolescents who never used cannabis but used e-cigarettes were more likely to use cannabis at follow-up than those living in states without RCL (aOR = 18.39, 95% CI: 4.25-79.68vs aOR = 5.09, 95% CI: 2.86-9.07, respectively) suggesting a risk of cannabis initiation among legal states.^38^ Among adolescents reporting recent alcohol and cannabis co-use, one study found a significant increase in the frequency of past 30-day cannabis use following RCL (*b* = 0.36, SE = 0.07, *P* ⩽ .001).^39^ In a Canadian study using a repeated cross-sectional design as well as a longitudinal design to examine changes in cannabis use, results revealed that adolescents had increased odds of ever using cannabis in the year following RCL in the cross-sectional data (*P* = .009).^40^ However, the longitudinal sample revealed no significant differences in the odds of ever use, current use, and regular use of cannabis post-legalization. There is also evidence of RCL impacts on adolescent cannabis use consequences, as a Washington study found a significant indirect effect of RCL on cannabis consequences through perceived risk as a mediator (*B* = 0.37, *P* ⩽ .001).^41^

On top of the above evidence, there were multiple studies examining cannabis use changes over time among adolescents in Washington and Oregon that found higher rates of cannabis use associated with cohorts examined during RCL compared to non-legal cohorts,^42-44^ although the differences across legal cohorts were not significant in all cases.^42^ Furthermore, in another study, RCL did not impact initiation of use, but for current users the RCL group had significantly greater increased rates of cannabis use compared to the pre-RCL group (RR = 1.26, 95% CI = 1.10, 1.45).^43^ For the final study, cannabis use increased in the post-RCL group but patterns of use (frequency; daily vs weekly use) were similar across groups.^44^ Overall, the preceding 8 studies reveal some evidence that RCL was associated with increasing rates of cannabis use in adolescent. However, 5 studies point to some inconsistent associations of RCL and cannabis use and suggest that overall relationship of RCL and adolescent cannabis as mixed.

Three studies add to these inconsistent findings and point to lack of an association between RCL and changes in cannabis use among adolescents. Two studies found no significant increase in the frequency of or prevalence of cannabis use following RCL.^41,45^ Finally, a study examining trends of adolescent cannabis use and associations with period effects (ie, external world events that could influence use) suggests laws and regulations associated with RCL were not associated with cannabis use changes.^46^ The current research reveals conflicting evidence about the role of RCL on adolescent cannabis use.

#### Cannabis use changes in young adults (~18-25)

Young adulthood, typically defined as ages 18 to 25 and also known as emerging adulthood, is commonly associated with decreased parental supervision, increased availability of substances, and greater substance experimentation making it a key developmental period for the onset of cannabis use.^47^ Four studies examined the impact of RCL on cannabis use among young adults, 2 of which found significant associations between RCL and increased cannabis use in college students.^47,48^ Barker and Moreno^48^ found the rate of students ever using cannabis did not change. However, in those who had used cannabis prior to RCL, the proportion of students using in the past 28-days increased faster following RCL in Washington (legal-state) when compared with the rate of increase in Wisconsin (non-legal state; *P* ⩽ .001).^48^ Further, in college students from Oregon, rates of cannabis use increased significantly from before to after RCL (*P* = .0002).^47^ Another study looked at changes in cannabis use in a sample of young adults from the U.S. who had never vaped cannabis at the time of recruitment.^49^ Results revealed that cannabis use in the past year did not differ in states with or without RCL, although, those living in states with RCL did show a larger increase in rates of cannabis vaping across time, compared to those in non-RCL states. Finally, in a sample of youth from Oregon and Washington, RCL predicted a higher likelihood of past-year cannabis use (*P* = .001).^50^ In contrast to the adolescent literature, studies examining cannabis use in young adult samples fairly consistently point to an association between RCL and increasing rates of cannabis use.

#### Cannabis use changes in general population adults

Five studies examined changes in cannabis use in adults (without further age subclassification) associated with RCL. Four of these studies suggested higher rates of cannabis use in adults for RCL jurisdictions compared to non-legal states post-RCL, or increased use following RCL.^37,45,51,52^ Past 30-day cannabis use increased significantly 1-month post-RCL and remained elevated 6-months post-RCL (ps = 0.01) in a sample of adults from California.^51^ Another study found an association between RCL and transition from non-users to cannabis users and non-users to weekly users when compared to states with no medical legalization or RCL (*P* ⩽ .001) and states with no legalization combined with those with medical cannabis legalization (*P* ⩽ .001).^37^ Meanwhile, in Canada, a significant increase in prevalence of cannabis use was observed following RCL.^45^ Additionally, in those reporting no cannabis use prior to RCL in Canada, there were significant increases in cannabis use frequency, quantity of cannabis used, and severity of cannabis misuse following RCL.^52^ The opposite pattern was seen for those reporting cannabis use prior to RCL, with significant decreases in frequency of use, quantity, and misuse.^52^ However, not all studies found RCL was associated with increased cannabis use. For instance, a repeated cross-sectional study of adult in the U.S. found no association between RCL and frequency of cannabis use.^53^

A benefit of the extant literature examining general population cannabis use is that it covers a variety of jurisdictions and study designs, albeit with some heterogeneity and mixed findings. On balance, the evidence within the current literature, generally suggests an increase in cannabis use for adults in the general population following RCL with 80% of the reviewed studies supporting this conclusion.

#### Maternal use

Three studies examined whether rates of cannabis use during pregnancy have increased following RCL. Two studies suggested increased cannabis use during pregnancy associated with RCL. In one study urine screen-detected cannabis use during pregnancy increased from 6% to 11% following RCL in California (*P* = .05).^54^ Another study in a sample of women participating in an intensive case management program for heavy alcohol and/or drug use during pregnancy, examined cannabis use among those exiting from the program before versus after RCL. Findings revealed women exiting after RCL were more likely to report using cannabis in the 30 days prior to exit compared to those pre-RCL (OR = 2.1, *P* ⩽ .0001).^55^ One study revealed no significant difference in cannabis or alcohol use associated with RCL in women living with HIV during pregnancy or the postpartum period.^56^ Overall, the evidence from these three studies suggests there may be increases in perinatal cannabis use following RCL, but the small number of studies and unique features of the samples suggests a need for more research.

#### Clinical populations use

Six studies examined cannabis use in clinical populations. One study investigated use and trauma admissions for adults and pediatric patients in California.^57^ Results showed an increase in adult trauma patients with THC+ urine tests from pre- to post-RCL (9.4% to 11.0%; *P* = .001), but no difference for pediatric trauma patients. A study based in Colorado and Washington, found that cannabis use rates in inflammatory bowel disease patients significantly increased from 107 users to 413 (*P* ⩽ .001) pre to post-RCL.^58^ A Canada-based study of women with moderate-to-severe pelvic pain found an increase in the prevalence of current cannabis use following RCL (13.3% to 21.5%; *P* ⩽ .001).^59^ Another Canadian study showed an increase in the prevalence of current cannabis use after RCL among cancer patients (23.1% to 29.1%; *P* ⩽ .01).^60^ Finally, two studies examined changes in cannabis use among individuals receiving treatment for a substance use disorder. In a sample of Canadian youth in an outpatient addictions treatment program, there was no change in the rate of cannabis use following RCL.^61^ Further, in a sample of individuals receiving treatment for opioid use disorder, cannabis use was compared for those recruited 6 months before or after RCL with no significant changes in the prevalence or frequency of self-reported (*P* = .348 and *P* = .896, respectively) or urine screen-detected (*P* = .087 and *P* = .638, respectively) cannabis use following RCL.^62^ Although these studies only represent a small number of observations, their findings do reveal associations between RCL and increasing cannabis use within some clinical samples.

#### Changes in polysubstance and other substance use

One study examined simultaneous cannabis and alcohol use among 7th, 9th, and 11th grade students in the U.S.^39^ This study found that RCL was associated with a 6% increase in the odds of past 30-day alcohol and cannabis co-use. The association was even stronger in students with past 30-day alcohol use and heavy drinking. However, among past 30-day cannabis users, RCL was associated with a 24% reduction in co-use. This study suggests at least a modest association between RCL and concurrent cannabis and alcohol use among adolescents.

Numerous studies examined changes of alcohol and other substance use pre to post RCL. With regard to alcohol, one study from Colorado and Washington found a decrease in alcohol consumption among adolescents following RCL,^42^ whereas another Washington study found RCL predicted a higher likelihood of alcohol use among youth.^50^ A Canadian study also found no significant effect of RCL on rates of alcohol or illicit drug use among youth.^61^ Finally, in a sample of trauma patients in California the findings around changes in substance use were mixed.^57^ In adult patients, the rates of positive screens for alcohol, opiates, methamphetamine, benzodiazepine/barbiturate, and MDMA did not change following RCL, but there was an increase in positive screens for cocaine. In pediatric patients, increases were seen in positive screens for benzodiazepine/barbiturate, but positive screens for alcohol, opiates, methamphetamine, and cocaine did not change.^57^ The current evidence is divided on whether RCL is associated with increased alcohol and other substance use, with 40% of studies finding an association and 60% not observing one or finding mixed results.

In the case of cigarettes, Mason et al^42^ did find significant cohort effects, where the post-RCL cohort was less likely to consume cigarettes compared to the pre-RCL one (Coefficient: − 2.16, *P* ⩽ .01). However, these findings were not echoed in more recent studies. Lack of an effect for cigarette use is supported by an Oregon study that found RCL was not associated with college student’s cigarette use.^47^ Similarly, RCL was not significantly associated with past-year cigarette use in a sample of young adults from Oregon and Washington.^50^ On balance, there is little evidence that RCL is linked with changes in cigarette smoking.

#### Route of administration

The increase in smoke-free alternative routes of cannabis administration (eg, vaping and oral ingestion of edibles)^63,64^ make method of cannabis consumption an important topic to understand in the context of RCL. Two studies examined differences in route of cannabis consumption as a function of cannabis policy. One study examined changes in the number of different modes of cannabis use reported by high school students in Canada.^65^ Results showed that from pre-to-post RCL 31.3% of students maintained a single mode of use, 14.3% continued to use cannabis in multiple forms, while 42.3% expanded from a single mode to multiple modes of administration and 12.1% reduced the number of modes they used. Another study found that smoking, vaping, and edibles (in that order) were the most frequent modes of cannabis use pre- and post-RCL in California, suggesting minimal impact of RCL on mode of cannabis use.^51^ However, the least common mode of cannabis use was blunts, which did decline following RCL (13.5%-4.3%).^51^ Overall, the evidence suggests RCL may be associated with changes in modes of cannabis consumption, but as the evidence is only from two studies there still remains a need for more studies examining RCL and cannabis route of administration.

### Attitudes

Nine studies examined RCL and cannabis attitudes (see Table 2). Regarding cannabis use intentions, one U.S. study found that for both a non-RCL state and a state that underwent RCL, intention to use in young adults significantly increased post-RCL, suggesting a lack of RCL specific effect,^48^ and that aside from the very first time point, there were no significant differences between the states in intention to use. Further, attitudes and willingness to use cannabis, between the RCL and non-RCL state remained similar overtime (*P*s ⩾ .05), although both states reported significantly more positive attitudes toward cannabis following RCL (*P* ⩽ .001).^48^ However, another study U.S. from found differences in adolescent use intentions across RCL, whereby those in the RCL cohort in jurisdictions that allowed sales were less likely to increase intent to use cannabis (*P* = .04), but the RCL cohort without sales were more likely to increase intent to use (*P* = .02).^43^ The pre-RCL cohort in communities that opted out of sales were also less likely to increase willingness to use compared to the cohort with legal sales (*P* = .02).^43^ Both studies reveal contrasting findings surrounding RCL’s relationship with cannabis use intentions and willingness to use.

**Table 2.** Studies examining recreational cannabis legalization and attitudes surrounding cannabis.

Author	Year	Location	Date of legalization	Study design	Sample	Brief findings
AminiLari et al	2022	Ontario	2018	Longitudinal	N = 254	Pre-RCL 25% of adults reported having medical cannabis authorization. Post-RCL the biggest shift in motivations for use was from solely medical to medical and recreational reasons. About ¼ of medicinal only users shifted to both medicinal and recreational reasons for use, and ¼ of participants reporting both reasons shifted to exclusively recreational reasons for use.
Bailey et al	2020	Washington	2012	Longitudinal	N = 281	RCL was not associated with perceived harm from cannabis use among youth.
Barker & Moreno	2021	Washington & Wisconsin	2012	Longitudinal cohort	N = 338	Attitudes toward cannabis were similar across states over time. However, post RCL attitudes toward cannabis became more positive for both states. Intentions to use cannabis also increased post RCL for both states.
Estoup et al	2016	Washington	2012	Cohort	N = 262	There was a significant indirect effect of RCL to cannabis-related consequences through lower perceived risk of use, but not frequency of use.
Gali et al	2021	California	2016	Longitudinal cohort	N = 429	Exposure to others cannabis use did not change post-RCL. Mental health perceptions from cannabis increased from slightly harmful to slightly beneficial. Physical health perceptions decreased 1-month post-RCL but increased 6-months post-RCL. Well-being perceptions remained similar 1-month post-RCL and increased 6-moths post-RCL.
Hawke & Henderson	2021	Ontario	2018	Cohort	N = 269	Reports of using cannabis alone, using with friends, and concealing cannabis use did not differ between the cohorts. Ease of cannabis access and reported safety of cannabis did not differ between cohorts.
Hawley et al	2019	British Columbia	2018	Repeated cross-sectional	N = 1673	The percent of cancer patients reporting some recreational reasons for cannabis increased post-RCL. Percent of pure medicinal users did decrease, non-significantly, post-RCL. Post-RCL cannabis users reported more problems accessing cannabis, with greatest barriers being lack of dispensaries and preferred products.
Rosic et al	2021	Ontario	2018	Repeated cross-sectional	N = 1390	The perceptions of how RCL would impact cannabis use did not change post- RCL. Most participants reported RCL would not/has no impact on use.
Rusby et al	2018	Oregon	2015	Cohort	N = 444	RCL cohort was more likely to increase cannabis intentions overtime, while the pre-RCL cohort was less likely to increase willingness and intent to use. RCL was not associated with initiating cannabis use.

Author, author of article; Year, publication year of article; Location, jurisdiction article data was collected in; Date of Legalization, year legalization was enacted in jurisdiction; Sample, total N of article sample; RCL, Recreational Cannabis Legalization.

Looking at cannabis use motives, one study found a non-significant increase in recreational motives for cannabis use post-RCL.^60^ Similarly following RCL in Canada, 24% of individuals previously reporting cannabis use exclusively for medical purposes declared using for both medical and non-medical purposes following RCL, and 24% declared use for non-medical purposes only,^66^ suggesting RCL can influence recreational/nonmedicinal motivations for cannabis use among those who previously only used for medical reasons.

In studies examining perceived risk and perceptions of cannabis use, one U.S. study found an indirect effect between RCL and increased consequences of use in adolescents through higher perceived risk (*P* ⩽ .001), but no association with frequency of use.^41^ Another U.S. study revealed mixed results and found that RCL was not associated with perceived harm of use in youth.^50^ Further, youth in one study did not report differences in perceptions of safety of cannabis, ease of accessing cannabis use or on concealing their use from authority,^61^ which contrasts with another study finding increased reports of problems accessing cannabis post-RCL (*P* ⩽ .01).^60^ Regarding health perceptions, a California study found that cannabis use was perceived as more beneficial for mental health, physical health, and wellbeing in adults at 6 months post-RCL compared to pre-RCL and 1-month post-RCL (*P* = .02).^51^ Mental health perceptions of cannabis use increased from being perceived as “slightly harmful” pre-RCL to perceived as “slightly beneficial” at 6 months post-RCL.^51^ However, in a sample of treatment seeking individuals with an opioid use disorder, the vast majority of participants reported beliefs that RCL would not impact their cannabis use, with no difference in beliefs pre- to post-RCL (85.9% reported belief it would have no impact pre-RCL and 85.7%, post-RCL).^62^ The combined results of the studies suggest potential associations of RCL with risk and benefit perceptions of cannabis use, however as 55% of studies suggest a lack of or inconsistent association with RCL, on balance the literature on RCL’s impact on cannabis attitudes is mixed.

### Health-related outcomes

We identified 33 articles that examined various health-related outcomes associated with RCL (see Table 3). The largest number involved hospital utilization (ie, seeking emergency services for cannabis-related problems such as unintentional exposure, CUD, and other harms). Other health-care outcomes included opioid-related harms, mental health variables, and adverse birth outcomes.

**Table 3.** Studies investigating the relationship of recreational cannabis legalization and health-related outcomes.

Author	Year	Location	Date of legalization	Study design	Sample	Brief findings
*Emergency service utilization*						
Baraniecki et al	2021	Ontario	2018	Retrospective chart review	N = 173	There was no difference in rate of cannabis intoxication related visits pre to post RCL. RCL was associated with an increase in patients 18 to 29. Post-RCL, the patients needing only observation increased, and the number of patients ordered for bloodwork or imaging decreased.
Calcaterra et al	2019	Colorado	2012	Archival administrative data	N = 38 406	Rates of cannabis related emergency visits significantly increased from 2009 to 2015. Alcohol related visits also increased, but to less of an extent than cannabis. Cannabis related emergency visits did show an abrupt increase following RCL.
Callaghan et al	2022	Alberta & Ontario	2018	Archival administrative data	N = 230 206	The rate of emergency department visits with cannabis-induced psychosis did not change pre- to post-RCL. Further, there was no change in admissions with amphetamine or alcohol induces psychosis.
Delling et al	2019	Colorado, New York, & Oklahoma	2012	Archival administrative data	Colorado: N = 2 088 909, New York: N = 11 726 283, Oklahoma: N = 2 334 988	The rate of change for cannabis diagnoses was greater in Colorado than New York and Oklahoma post-RCL. There were decreased admissions for cannabis abuse in Colorado compared to Oklahoma post-RCL. Healthcare costs and length of patient stay showed no significant difference across state. Colorado also had increased motor vehicle accidents, alcohol abuse, injection overdose injuries, and decreased chronic pain admissions post-RCL compared to both states.
Grigorian et al	2019	California	2016	Archival administrative data	N = 21 173	Post-RCL also had significantly higher adult trauma activation. Both adults and pediatrics had increased mortality rates post-RCL.
Kim et al	2022	Ontario	2018	Interrupted time series	N = 14 900 820	Cannabis-related emergency department visits increased for individuals under 65 post-RCL. RCL was associated with immediate visits for men 45 to 64, women 25 to 44, and women 45 to 65. However, RCL was not associated with trend level increases in emergency visits.
Masonbrink et al	2021	U.S.		Cohort	N = 1 898 432	RCL was associated with increased adolescent cannabis-related admissions from 2008 to 2019. While there was an increasing trend pre-RCL, the rate of increase in admissions accelerated post-RCL.
Mennis & Stahler	2020	Colorado & Washington	2012	Archival administrative data	N = 653 232	Adolescent cannabis treatment admissions rates decreased in both states over time, with steep declines post-RCL. The decrease in admissions for both states was greater than non-legal states but not significantly.
Myran et al	2022	Ontario	2018	Repeated cross-sectional	N = 13 853 396	Cannabis-related emergency visits in youth and young adults were increasing pre-RCL, but RCL was associated with an immediate spike followed by a monthly attenuation in rate of visits.
Myran et al	2022	Ontario	2018	Repeated cross-sectional	N = 14 375 697	Rates of cannabis hyperemesis related emergency visits were increasing pre-RCL. Post-RCL there was no significant change in rates of emergency visits, but the increasing trend continued.
Myran et al	2022	Canada	2018	Archival administrative data	N-581	Children hospital admissions for cannabis poisonings increased 2.6x post-RCL for all provinces examined (British Columbia, Alberta, Ontario, Quebec).
Pusateri et al	2022	Colorado & Washington	2012	Archival administrative data	N = 18 545	Rates of steroid use and need for total parenteral nutrition in irritable bowel disease patients decreased post-RCL. Total hospital costs in patients also dropped post-RCL. In cannabis users specifically, there was less patients needing total parenteral nutrition and lower hospital costs post-RCL.
Roth et al	2022	California	2016	Archival administrative data	N = 12 108	Post-RCL monthly cannabis-exposure poisons control calls significant increased. By age, exposures in youth under 13 significant increase post-RCL, but there was no change for those 13+.
Sokoya et al	2018	Colorado	2012	Archival administrative data	N = 2164	There was no change in number of facial fractures pre to post RCL. Maxillary and skull base fractures were the only type to significantly increase post-RCL.
Thomas et al	2019	Washington	2012	Archival administrative data	N = 161	The number of unintentional pediatric cannabis exposures per month increased post-RCL.
Wang et al	2018	Colorado	2012	Archival administrative data	N = 4202	Overall, 67% of adolescent patients had THC positive urine drug screens. The rate of annual cannabis-related visits to emergency care significantly increased over time. Behavioral health evaluations from visits also increased over time.
Wang et al	2022	Colorado	2012	Archival administrative data	N = 262 699	Cannabis-related pregnancy admissions significantly increased from 2011 to 2018, with spikes in 2012 and 2014.
Wang et al	2017	Colorado	2012	Archival administrative data	N = 7 440 392	Cannabis related hospitalizations increased over time, with the greatest increases in 2009 and 2014. Visits associated with mental illness were more common in cannabis related visits. Poison control calls remained stable but there were significant increases in 2010. There were increases in calls for those under 17 and over 25 after 2014. Unintentional cannabis exposure increased for those 0 to 8 from 2008 to 2014 and for 9+ year old’s from 2013 to 2015.
Wang et al	2016	Colorado	2012	Archival administrative data	N = 244	Unintentional cannabis exposure in children increased 2 years post-RCL compared to 2 years pre-RCL. There was also a significant increase in poison control cases over time. This increase was significantly greater compared to the rest of the U.S.
Yeung et al	2021	Alberta	2018	Archival administrative data	N = 1920	Overall pediatric cannabis-related emergency department visits did not change pre- to post-RCL. For specific age groups rate and proportion of visits for children under 12 increased post-RCL. Emergency visit rates for cannabis and other substances decreased in adolescents 15 to 17. For cannabis co-diagnoses, the proportion of cannabis hyperemesis presentations increased post-RCL in adolescents 15 to 17. Unintentional cannabis ingestion rates did increase post-RCL for children and older adolescents, but not for younger adolescents.
Yeung et al	2020	Alberta	2018	Archival administrative data	N = 14 732	The volume of cannabis-related emergency department visits and poison control calls increased post-RCL. Cannabis and other substance admissions and co-diagnoses decreased post-RCL.
*Opioid use*						
Dranitsaris et al	2021	Canada	2018	Archival administrative data	Public and private prescription claims	There was a steady decline in volume of opioids prescribed for public and private drug plans. Post-RCL there was a significant spike in the rate of declines (5.4x greater than pre-RCL).
Geoffrion et al	2021	British Columbia	2018	Archival administrative data	N = 3705	Post-RCL women were less likely to consume opioids and other narcotics.
Livingston et al	2017	Colorado	2012	Interrupted time series	CDC and Prevention WONDER from 2000 to 2015	There was a significant decrease in opioid-related deaths post-RCL. Even after controlling for trends in comparison states there was still a significant reduction.
Lopez et al	2021	U.S.		Archival administrative data	N = 144 000	There was no significant association between RCL and opioid prescriptions by an orthopedic surgeon. RCL states had non-significant increases in daily doses of opioid and hydrocodone prescriptions respectively.
Shi et al	2019	U.S.		Archival administrative data	Medicaid State Drug Utilization Data	RCL states had slightly greater, not significantly, Schedule II and III opioid prescriptions compared to medical only states. States with RCL in 2015 to 2017 had reduced Schedule III prescriptions while states with RCL to 2012 had increases. RCL was not associated with number of prescriptions, total doses, or spending of Schedule II opioids. However, RCL in 2015 was associated with the former two and Schedule III spending.
*Adverse birth outcomes*						
Siega-Riz et al	2020	Colorado & Washington	2012	Archival administrative data	N = 1 347 916	The rate of small for gestational age births did not change pre to post RCL in both Washington and Colorado. Pre-term births did increase post-RCL but only in Colorado. Congenital anomalies significantly increased for both states pre to post-RCL.
Straub et al	2021	Washington	2012	Archival administrative data	N = 5343	The prevalence of positive THC screens in women giving birth did not change over time. The prevalence of low-birth-weight births did increase from pre to post-RCL. However, RCL was not associated with small for gestational age births.
*Mental health outcomes*						
Callaghan et al	2022	Alberta & Ontario	2018	Archival administrative data	N = 230 206	Emergency visits with schizophrenia and related conditions codes did not change pre- to post-RCL.
Geoffrion et al	2021	British Columbia	2018	Archival administrative data	N = 3705	Post-RCL, women had higher anxiety scores than pre-RCL.
Hawke & Henderson	2021	Ontario	2018	Cohort	N = 269	There were no significant differences for the pre and post-RCL cohorts for internalizing or externalizing disorders or crime/violence screenings.
Rusby et al	2019	Oregon	2014	Ecological momentary assessment	N = 466	Cannabis users had higher mood lability scores compared to non-users. RCL had no impact on the association of anxious mood and cannabis use.
Vignault et al	2021	Quebec	2018	Archival administrative data	N = 2615	Prevalence of psychotic disorders did not differ pre- to post-RCL, but personality disorders and other psychiatric disorders were more prevalent post-RCL.
Yeung et al	2021	Alberta	2018	Archival administrative data	N = 1920	Personality and mood related co-diagnosis decreased post-RCL for adolescents 15 to 17.
*Miscellaneous health outcomes*						
Fedorova et al	2022	California	2016	Longitudinal	N = 668	Approximately half of medical cannabis patients remained so from pre- to post-RCL. The most common transition group pre- to post-RCL was out of medical cannabis patient status, followed by never been issued a medical cannabis recommendation, with into medical cannabis patient at the smallest transition group. RCL was the most common reason reported for transitioning out if medical cannabis patient status.
Geoffrion et al	2021	British Columbia	2018	Archival administrative data	N = 3705	Post-RCL, women had higher pain catastrophizing scores than pre-RCL. Post-RCL women were less likely to consume anti-inflammatories, and nerve medications to treat pelvic pain, but more likely to use herbal pain medication.
Jordan et al	2022	New Brunswick	2018	Retrospective chart review	N = 3060	The proportion of post-mortem positive cannabis screens did increase from pre- to post-RCL but was not significant following Benjamini-Hochberg correction. The only age group with a significant increase in proportion of positive screens post-RCL was 25-44-year-olds. Those who died post-RCL did have higher odds of cannabis present post-mortem. Tests for cannabinoid detection, did find an increase in positive detection over time, with the steepest increases occurring pre-RCL. There was no change in detection of other drugs.

Author, Author of article; Year, Publication year of article; Location, Jurisdiction article data was collected in; Date of Legalization, Year legalization was enacted in jurisdiction; Sample, Total N of article sample; CDC, Center for Disease Prevention; WONDER, Wide-Ranging Online Data for Epidemiologic Research; RCL, Recreational Cannabis Legalization.

#### Emergency service utilization

Seventeen studies examined the association between RCL and use of emergency services related to cannabis (eg, hospital visits, calls to regional poison centers). Regarding emergency service rates in youth, a Colorado study found the rate of pediatric cannabis-related emergency visits increased pre- to post-RCL (*P* ⩽ .0001).^67^ Similarly, cannabis-related visits requiring further evaluation in youth also increased.^67^ This increasing need for emergency service related to cannabis exposure in youth following RCL was supported in 4 other U.S. studies.^68-71^ A Canadian study supported the U.S. studies, finding a 2.6 increase in children admissions for cannabis poisonings post-RCL.^72^ In contrast, overall pediatric emergency department visits did not change from pre- to post-RCL in Alberta, Canada,^73^ but there was a non-significant increase of the rate and proportion of children under 12 presenting to the emergency department. However, unintentional cannabis ingestion did increase post-RCL for children under 12 (95% CI: 1.05-1.47) and older adolescents (1.48, 95% CI: 1.21-1.81).^74^ Taken together, these studies do suggest a risk for increasing cannabis-related emergency visits in youth following RCL, with 75% of studies finding an association between RCL and increasing emergency service rates in youth.

There is also evidence of increased hospital utilization in adults following RCL. Five studies found evidence of increased emergency service utilization or poison control calls from cannabis exposure associated with RCL in the U.S. and Canada.^24,69,74-76^ Finally, a Colorado study saw an increase in cannabis involved pregnancy-related hospital admissions from 2011 to 2018, with notable spikes after 2012 and 2014, timeframes associated with state RCL.^77^

However, some evidence points to a lack of association between RCL and emergency service utilization. A chart review in Ontario, Canada found no difference in number of overall cannabis emergency room visits pre- versus post-RCL (*P* = .27).^78^ When broken down by age group, visits only increased for those 18 to 29 (*P* = .03). This study also found increases in patients only needing observation (*P* = .002) and fewer needing bloodwork or imaging services (both *P*s ⩽.05).^78^ Further in a California study that found overall cannabis exposure rates were increasing, when breaking these rates down by age there was no significant change in calls for those aged 13 and up, only for those 12 and under.^69^ An additional Canadian study found that rates of cannabis related visits were already increasing pre-RCL.^79^ Following RCL, although there was a non-significant immediate increase in in cannabis-related emergency visits post-RCL this was followed a significant drop off in the increasing monthly rates seen prior to RCL.^79^ Another Canadian study that examined cannabis hyperemesis syndrome emergency visits found that rates of admissions were increasing prior to RCL and the enactment of RCL was not associated with any changes in rates of emergency admissions.^80^ As this attenuation occurred in Canada prior to commercialization where strict purchasing policy was in place, it may suggest that having proper regulations in place can prevent the uptick in cannabis-related emergency visits seen in U.S. studies.

Other hospital-related outcomes examined included admissions for cannabis misuse and other substance use exposure. One study found decreasing CUD admission rates over time (95% CI: −4.84, −1.91), with an accelerated, but not significant, decrease in Washington and Colorado (following RCL) compared to the rest of the U.S.^81^ In contrast, another study found increased rates of healthcare utilization related to cannabis misuse in Colorado compared to New York and Oklahoma (*P*s ⩽.0005).^82^ With respect to other substance use, findings revealed post-RCL increases in healthcare utilization in Colorado for alcohol use disorder and overdose injuries but a decrease in chronic pain admissions compared to both controls (*P* ⩽ .05).^82^ However, two Canadian studies found the rate of emergency department visits with co-ingestant exposure of alcohol, opioid, cocaine, and unclassified substances in older adolescents and adults decreased post-RCL.^73,77^ Another Canadian study found no change in cannabis-induced psychosis admissions nor in alcohol- or amphetamine-induced admissions.^83^

Finally, three studies examined miscellaneous hospital-related outcomes. A study examining hospital records in Colorado to investigate facial fractures (of significance as substance impairment can increase the risk of accidents) showed a modest but not significant influence of RCL.^84^ The only significant increases of facial trauma cases were maxillary and skull base fracture cases (*P*s ⩽ .001) suggesting a partial influence of RCL on select trauma fractures. The second study found increased trauma activation (need for additional clinical care in hospital) post-RCL in California (*P* = .01).^57^ Moreover, both adult and pediatric trauma patients had increased mortality after RCL (*P* = .03; *P* = .02, respectively).^57^ The final study examining inflammatory bowel disease (IBD) outcomes in the U.S. found more cannabis users on total parenteral nutrition post-RCL (95% CI: 0.02, 0.89) and lower total hospital costs in users post-RCL (95% CI: −15 717, −1119).^58^ No other IBD outcomes differed pre- to post-RCL (eg, mortality, length of stay, need for surgery, abscess incision and drainage).

Overall, these studies point to increased cannabis-related health-care utilization following RCL for youth and pediatrics (75% finding an increase). However, the impact of legalization on adult rates of cannabis-related emergency visits is mixed (44% finding lack of an association with RCL). As findings also varied across different countries (ie, Canada vs the U.S.), it suggests the importance of continually monitoring the role of RCL across different jurisdictions which may have different cannabis regulations in place. These studies also suggest there may be other health consequences associated with RCL. Further research should be done to examine trends of other emergency service use that could be influenced by RCL.

#### Opioid use

Two studies reported a weak or non-existent effect of RCL on opioid related outcomes.^85,86^ First, a U.S. administrative study found no association of RCL and opioid prescriptions from orthopedic surgeons.^85^ The second study found that, of U.S. states that passed RCL, those that passed policies before 2015 had fewer Schedule III opioid prescriptions (*P* = .003) and fewer total doses prescribed (*P* = .027),^86^ but when compared to states with medicinal cannabis legislation, there were no significant differences. However, 3 studies suggested a potential protective effect of RCL, with one study finding a significant decrease for monthly opioid-related deaths following RCL (95% CI: –1.34, –0.03), compared to medical cannabis legalization and prohibition.^87^ A Canadian study examining opioid prescription claims also found an accelerated decline in claims for public payers post-RCL compared to declines seen pre-RCL (*P* ⩽ .05).^88^ Next a study examining women with pelvic pain found that post-RCL patients were less likely to report daily opioid use, including use for pain (*P* = .026).^59^ These studies indicate some inconsistencies in relationships between RCL, opioid prescriptions and use indicators in the current literature, while the literature on balance points to a potential relationship with RCL (60%), the overall evidence is still mixed as 40% of studies support a weak association with RCL.

#### Adverse birth outcomes

Changes in adverse birth outcomes including small for gestational age (SGA) births, low birth weight, and congenital anomalies were examined in two studies. The first study, which examined birth outcomes in both Colorado and Washington, found that RCL was associated with an increase in congenital anomaly births for both states (*P* ⩽ .001, *P* = .01 respectively).^89^ Preterm births also significantly increased post-RCL, but only in Colorado (*P* ⩽ .001). Regarding SGA outcomes, there was no association with RCL for either state.^89^ Similarly, the second study did find an increase in the prevalence of low birth weight and SGA over time, but RCL was not directly associated with these changes.^90^ Although the current literature is small and limited to studies in Washington and Colorado, the evidence suggests minimal changes in adverse birth outcomes following RCL.

#### Mental health outcomes

Six studies examined mental health related outcomes. A Canadian study examining psychiatric patients did not see a difference in rates of psychotic disorders pre- to post-RCL.^45^ Similarly, another Canadian study did not see a difference in hospital admissions with schizophrenia or related codes post-RCL.^83^ However, the prevalence of personality disorders and “other” diagnoses was higher post-RCL (*P* = .038).^45^ In contrast, another Canadian study found that rates of pediatric cannabis-related emergency visits with co-occurring personality and mood-related co-diagnoses decreased post-RCL among older adolescents.^73^ A U.S. study examining the relationship between cannabis use and anxious mood fluctuations in adolescents found RCL had no impact on the association.^91^ Similarly, another Canadian study found no difference in mental health symptomology pre- to post-RCL.^61^ In contrast, anxiety scores in women with pelvic pain were higher post-RCL compared to pre-RCL (*P* = .036).^59^ The small number and mixed findings of these studies, 66.7% finding no association or mixed findings and 33.3% finding an association but in opposite directions, identify a need for further examination of mental health outcomes post-RCL.

#### Miscellaneous health outcomes

Three studies examined additional health-related outcomes. First, a California study examined changes in medical cannabis status across RCL. Post-RCL, 47.5% of medical cannabis patients remained medical cannabis patients, while 73.8% of non-patients remained so.^92^ The transition into medical cannabis patient status post-RCL represented the smallest group (10%). Cannabis legalization was the most reported reason for transition out of medical cannabis patient status (36.2%).^92^ Next, a study examining pelvic pain in women found that post-RCL patients reported greater pain catastrophizing (*P* ⩽ .001), less anti-inflammatory (*P* ⩽ .001) and nerve medication use (*P* = .027), but more herbal pain medication use (*P* = .010).^59^ Finally, a Canadian study that examined cannabinoids in post-mortem blood samples reported that post-RCL deaths had higher odds of positive cannabis post-mortem screens compared to pre-RCL (95% CI: 1.09-1.73).^93^ However, the majority of growth for positive cannabinoid screens took place in the two years prior to RCL implementation. In sub-group analyses, only 25- to 44-year-olds had a significant increase in positive cannabinoid screens (95% CI: 0.05-0.19). Additional post-mortem drug screens found an increase in positive screens for amphetamines (*P* ⩽ .001) and cocaine (*P* = .042) post-RCL. These additional health outcomes demonstrate the wide-ranging health impacts that may be associated with RCL and indicate a continued need to examine the role of RCL on a variety of outcomes.

### Driving-related outcomes

Six studies examined rates of motor vehicle accidents and fatalities (see Table 4). Two U.S. studies found no statistical difference in fatal motor vehicle collisions associated with RCL.^94,95^ Further, a California-based study examining THC toxicology screens in motor vehicle accident patients, did find a significant increase in positive screens, but this increase was not associated with implementation of RCL.^96^ However, three studies suggest a negative impact of RCL, as one U.S. study found both RCL states and their neighboring states had an increase in motor vehicle fatalities immediately following RCL.^97^ Additionally, a Canadian study did find a significant increase in moderately injured drivers with cannabis positive blood screens post-RCL.^98^ Finally, a study in Uruguay found RCL was associated with increased immediate fatal crashes for cars, but not motorcycles; further investigation suggested this effect was noticeable in urban areas, but not rural areas.^99^ While the overall evidence was inconsistent, current evidence does suggest a modest increase, seen in two studies, in motor vehicle accidents associated with RCL. Further longitudinal research in more jurisdictions is needed to understand the long-term consequences of RCL on motor vehicle accidents.

**Table 4.** Studies looking at recreational cannabis legalization and driving related outcomes.

Author	Year	Location	Date of legalization	Study design	Sample	Brief findings
Aydelotte et al	2017	Colorado & Washington	2012	Archival administrative data	N = 60 737	Rates of fatal car crashes did not differ between both states pre-RCL and controls. Post-RCL, there were no significant changes in fatality rates.
Aydelotte et al	2019	Colorado & Washington	2012	Archival administrative data	N = 25 561	Rates of fatal accidents were non-significantly higher in both states post-RCL than control states.
Borst et al	2021	California	2016	Archival administrative data	N = 11 491	The rate of drivers testing positive for cannabis over time did increase. However, there was not a significant association with RCL, suggesting that the increasing rates were not driven by RCL.
Brubacher et al	2022	British Columbia	2018	Archival administrative data	N = 4339	There was a significant increase in moderately injured drivers testing positive for THC with a THC level of 2 ng/ml and 5 ng/ml post-RCL.
Lane & Hall	2018	Colorado, Washington & Oregon	2012	Interrupted time series	CDC and Prevention WONDER	There was significant increase in traffic fatalities post-RCL. Neighboring states of Colorado also had significant increases in followed significant trend reductions, suggesting RCL creates a temporary increase in fatalities.
Nazif-Munoz et al	2020	Uruguay	2013	Interrupted time series	National Road Safety Agency of Uruguay and the Ministry of Transport and Public Work	RCL was associated with an immediate increase in light motor vehicle driver fatality rate in larger cities. However, there was no change in light motor vehicle driver fatality rates in rural areas. There was no significant change associated with RCL for motorcyclist fatality rates.

Author, Author of article; Year, Publication year of article; Location, Jurisdiction article data was collected in; Date of Legalization, Year legalization was enacted in jurisdiction; Sample, Total N of article sample; CDC, Center for Disease Prevention; WONDER, Wide-Ranging Online Data for Epidemiologic Research; RCL, Recreational Cannabis Legalization.

### Crime-related outcomes

Three studies explored crime-related outcomes associated with RCL (see Table 5). A Washington study examining cannabis-related arrest rates in adults did find significant drops in cannabis-related arrests post-RCL for both 21+ year olds (87% drop; *P* ⩽ .001) and 18 to 20-year-olds (46% drop; *P* ⩽ .001).^100^ However, in another study examining Oregon youth this post-RCL decline for arrests was not seen; cannabis-related allegations in youth actually increased following RCL (28%; 95% CI = 1.14, 1.44).^101^ Further, declines in youth allegations prior to RCL ceased after RCL was implemented. In contrast, a Canadian study did find significant decreases in cannabis-related offenses in youth post RCL (*P* ⩽ .001), but rates of property and violent crime did not change across RCL.^102^ These studies highlight the diverse effects of RCL across different age groups. However, there remains a need for a more comprehensive evaluation on the role of RCL on cannabis-related arrests.

**Table 5.** Studies investigating recreational cannabis legalization and crime related outcomes.

Author	Year	Location	Date of legalization	Study design	Sample	Brief findings
Callaghan et al	2021	Canada	2018	Archival administrative data	N = 32 178	RCL was associated with a significant decrease in daily cannabis-related offenses in youth overall and when broken down by sex. There was no evidence of an RCL association for property or violent crime rates in youth.
Firth et al	2020	Oregon	2014	Interrupted time series	N = 18 779	Overall rate of cannabis-related allegations increased post-RCL. American Indian/Alaskan Native more likely than White youth to have an allegation pre-and post-RCL but was stable over time. Black youth also more likely than White youth pre-RCL with the disparity decreasing post-RCL.
Firth et al	2019	Washington	2012	Archival administrative data	National Incident Based Reporting System 2012-2015	Arrest rates dropped in those 21+ after post-RCL. Arrest rates for 18 to 20 decreased post-RCL. Rates for Black individuals 21+ dropped post-RCL but relative disparities from White individuals increased. Rates for Black individuals 18 to 20 also dropped post-RCL but there was no significant increase in relative disparities to White counterparts. Arrest rates for selling cannabis did drop more for White individuals compared to Black individuals.

Author, Author of article; Year, Publication year of article; Location, Jurisdiction article data was collected in; Date of Legalization, Year legalization was enacted in jurisdiction; Sample, Total N of article sample; RCL, Recreational Cannabis Legalization.

Notably, two studies also examined race disparities in cannabis-related arrests. For individuals 21+ relative arrest disparities between Black and White individuals grew post-RCL.^100^ When looking at 18 to 20-year-olds, cannabis-related arrest rates for Black individuals did slightly decrease, albeit non-significantly, but there was no change in racial disparities.^100^ In youth ages 10 to 17, Indigenous and Alaska Native youth were more likely than White youth to receive a cannabis allegation before RCL (95% CI: 2.31, 3.01), with no change in disparity following RCL (95% CI: 2.10, 2.81).^101^ On the other hand, Black youth were more likely to receive a cannabis allegation than White youth prior to RCL (95% CI: 1.66, 2.13), but the disparity decreased following RCL (95% CI: 1.06, 1.43).^101^ These studies suggest improvements in racial disparities for cannabis-related arrests following RCL, although there ware only two studies and they are limited to the U.S.

## Discussion

The aim of this systematic review was to examine the existing literature on the impacts of RCL on a broad range of behavioral and health-related outcomes. The focus on more rigorous study designs permits greater confidence in the conclusions that can be drawn. The literature revealed five main outcomes that have been examined: cannabis use behaviors, cannabis attitudes, health-related outcomes, driving-related outcomes, and crime-related outcomes. The overall synthesizing of the literature revealed heterogenous and complex effects associated with RCL implementation. The varied findings across behavioral and health related outcomes does not give a clear or categorical answer as to whether RCL is a negative or positive policy change overall. Rather, the review reveals that while a great deal of research is accumulating, there remains a need for more definitive findings on the causal role of RCL on a large variety of substance use, health, attitude-related, driving, and crime-related outcomes.

Overall, studies examining cannabis use behavior revealed evidence for cannabis use increases following RCL, particularly for young adults (100%), peri-natal users (66%), and certain clinical populations (66%).^47,54,59^ While general adult samples had some mixed findings, the majority of studies (80%) suggested increasing rates of use associated with RCL.^51^ Of note, the increasing cannabis use rates found in peri-natal and clinical populations are particularly concerning as they do suggest increasing rates in more vulnerable samples where potential adverse consequences of cannabis use are more pressing.^103^ However, for both groups the overall literature revealed only a few studies and thus requires further examination. Further, a reason to caution current conclusions surround RCL impacts on substance use, is that there is research suggesting cannabis use rates were increasing prior to RCL in Canada.^104^ Thus, there still remains a need to better disentangle causal consequences of RCL on cannabis use rates.

In contrast to studies of adults, studies of adolescents pointed to inconsistent evidence of RCL’s influence on cannabis use rates,^38,45^ with 60% of studies finding no change or inconsistent evidence surrounding adolescent use following RCL. Thus, a key conclusion of the cannabis use literature is that there is not overwhelming evidence that RCL is associated with increasing rates of cannabis among adolescents, which is notable as potential increases in adolescent use is a concern often voiced by critics of RCL.^16^ This might suggest that current RCL policies that limit access to minors may be effective. However, a methodological explanation for the discrepancy between findings for adolescents and adults is that adults may be more willing to report their use of cannabis following RCL as it is now legal for them to use. However, for adolescents’ cannabis use remained illicit, which may lead to biased reporting from adolescents. Thus, additional research using methods to overcome limitations of self-reports may be required.

With regard to other substance use, primarily alcohol and cigarettes, there is little evidence that RCL is associated with increased use rates and may even be associated with decreased rates of cigarette use.^42,61^ The lack of a relationship with RCL and increasing alcohol and other substance use, seen in 60% of studies, is relevant due to concerns of RCL causing “spill-over” effects to substances other than cannabis. However, the decreasing rates on cigarette use associated with RCL seen in 33% of studies may also suggest a substitution effect of cannabis.^105^ It is possible that RCL encourages a substitution effect where cannabis is used to replace use other substances such as cigarettes, but 66% of studies found no association of RCL and cigarette use so further research examining a potential substitution effect is needed. In sum, the literature points to a heterogenous impact of RCL on cannabis and other substance use rates, suggesting complex effects of RCL on use rates that may vary across age and population. However, the review also highlights that there are still limited studies examining RCL and other substance use, particularly a lack of multiple studies examining the same age group.

The current evidence for the impact of RCL on attitudes surrounding cannabis revealed mixed or limited results, with 44% studies finding some sort of relationship with attitudes and RCL and 55% studies suggest a lack of or inconsistent relationship. Studies examining cannabis use attitudes or willingness to use revealed conflicting evidence whereas some studies pointed to increased willingness to use associated with RCL,^43^ and others found no change or that changes were not specific to regions that implemented RCL.^48^ For attitude-related studies that did reveal consistent findings (eg, use motivation changes, perceptions of lower risk and greater benefits of use), the literature was limited in the number of studies or involved heterogenous samples, making it difficult to make conclusive statements surrounding the effect of RCL. As cannabis-related attitudes (eg, perceived risk, intentions to use) can have implications for cannabis use and consequences^106,107^ it is interesting that current literature does not reveal clear associations of cannabis-related attitudes and RCL. Rather, this review reveals a need for more research examining changes in cannabis-attitudes over time and potential impacts of RCL.

In terms of health outcomes, the empirical literature suggests RCL is associated with increased cannabis-related emergency visits^24,67,70,76^ and other health consequences (eg, trauma-related cases^57^). The literature also suggests there may be other potential negative health consequences associated with RCL, such as increasing adverse birth outcomes and post-mortem cannabis screens.^45,89^ Synthesizing of the literature points to a well-established relationship of RCL and increasing cannabis-related emergency visits. While some extant literature was mixed, on balance most studies included in the review (70.6%) found consistent evidence of increased emergency service use (eg, emergency department admissions and poison control calls) for both adolescents and adults with only 31% of studies finding mixed or no association with RCL. This points to a need for stricter RCL policies to prevent unintentional consumption or hyperemesis such as promoting safe or lower risk use of cannabis (eg, using lower THC products, avoiding deep inhales while smoking), clearer packaging for cannabis products, and safe storage procedures.

However, the literature on health outcomes outside of emergency service utilization is limited and requires more in-depth evaluations to be fully understood. Additionally, not all health-outcomes indicated negative consequences associated with RCL. There is emerging evidence of the potential of RCL to help decrease CUD and multiple substance hospital admissions^74,82^ Furthermore, while some findings were mixed and the number of studies limited, 60% of studies found potential for RCL to have protective effects for opioid-related negative consequences.^87,88^ However, opioid-related findings should be considered in the context of population-level changes in opioid prescriptions and shifting opioid policy influence.^108^ Thus, findings may be a result of changes driven by the response to the opioid epidemic rather than RCL, and there remains a need to better disentangle RCL impacts on opioid-related consequences. It is also worth noting that some opioid and cannabis studies are underwritten by the cannabis industry, so the findings should be interpreted with caution due to potential for conflicts of interest.^88^ In sum, the overall literature suggests that RCL is associated with both negative and positive health-related consequences and reveals a need to examine the role of RCL across a wide range of health outcomes.

The findings from the driving-related literature do suggest RCL is associated with increased motor vehicle accidents (50% of studies) although the literature was quite evenly split as higher accident rates were not seen across all studies (50% studies). These results point to potential negative consequence associated with RCL and may indicate a need for better measures to prevent driving while under the influence of cannabis in legalized jurisdictions. However, as the evidence was split and predominately in the U.S. additional studies spanning diverse geographical jurisdictions are still needed.

On the other hand, the findings from crime-related outcomes showed some inconsistencies. While one study did suggest minimal decreases for substance-use related arrests in adults, the findings were not consistent across the two studies examining arrest-rates in youth.^100-102^ These potential decreases in arrest rates for adults can have important implications as cannabis-related crime rates make up a large amount of overall crime statistics and drug-specific arrests.^30,31^ This discrepancy in youth findings between a U.S. and Canadian study are notable as Canadian RCL policies do include stipulations to allow small scale regulations in youth. Thus, it suggests RCL policies that maintain prohibition of use among underage youth do not address issues related to arrests and crime among youth. In fact, the current literature suggests that cannabis-related charges are still being enforced for youth under the legal age of consumption in the U.S. Another important outcome revealed is racial disparities in cannabis-related arrests. Previous evidence has shown there are racial disparities, particularly between Black, Indigenous, and Hispanic individuals compared to White counterparts, in cannabis-related charges and arrests.^109,110^ Regarding racial disparities and RCL, there was very little evidence of decreases in disparities for cannabis-related arrests following RCL.^100,101^ This racialized arresting is significant as it can be associated with additional public health concerns such as physical and mental health outcomes, harm to families involved, and to communities.^111^ This finding is particularly concerning as it suggests racialized arrests for cannabis are still occurring despite the intentions of liberalization of cannabis policies to help reduce racial disparities in the criminal justice system. However, it is important to note that there were only 2 studies of racial disparities in cannabis-related arrests and both were conducted in the U.S. Thus, additional research is required before drawing any firm conclusions about the ability of RCL to address systemic issues in the justice system.

### Limitations

The findings should be considered within context of the following limitations. The research was predominately from North America (U.S. and Canada). While both countries have either federal or state RCL, findings only from two countries that are geographically connected may not reflect the influence of RCL across different cultures and countries globally. The majority of studies also relied on self-report data for cannabis-related outcomes. Thus, there is a risk that any increases in use or other cannabis-related outcomes may be due to an increased comfort in disclosing cannabis use due to RCL.

Given the large number of studies on multiple outcomes, we chose to focus on implementation of RCL exclusively, rather than related policy changes such as commercialization (ie, the advent of legal sales), to allow for clearer conclusions about the specific impacts on RCL. However, a limitation is that the review does not address the impact of commercialization or changes in product availability. While outside the scope of the current review, it does limit the conclusions that can be drawn about RCL overall as some jurisdictions implemented features of commercialization separately from legalization. For example, in Ontario, Canada, storefronts and edible products became legal a year after initial RCL (when online purchase was the exclusive modality), which may have had an additional impact on behavioral and health-related outcomes. Additionally, the scope of the review was limited to recreational legalization and did not consider other forms of policy changes such as medicinal legalization or decriminalization, as these have been summarized more comprehensively in prior reviews.^112-114^ Further, this review focused on behavioral and health outcomes; other important outcomes to examine in the future include economic aspects such as cannabis pricing and purchasing behaviors, and product features such as potency. Finally, as this review considered a broad range of outcomes, we did not conduct a meta-analysis which limits conclusions that can be drawn regarding the magnitude of the associations.

## Conclusions

The topic of RCL is a contentious and timely issue. With nationwide legalization in multiple countries and liberalizing policies across the U.S., empirical research on the impacts of RCL has dramatically expanded in recent years. This systematic review comprehensively evaluated a variety of outcomes associated with RCL, focusing on longitudinal study designs and revealing a wide variety of findings in terms of substance use, health, cannabis attitudes, crime, and driving outcomes examined thus far. However, the current review highlights that the findings regarding the effects of RCL are highly heterogenous, often inconsistent, and disproportionately focused on certain jurisdictions. With polarizing views surrounding whether RCL is a positive or negative policy change, it is noteworthy that the extant literature does not point to one clear answer at the current time. In general, the collective results do not suggest dramatic changes or negative consequences, but instead suggest that meaningful tectonic shifts are happening for several outcomes that may or may not presage substantive changes in personal and public health risk. Furthermore, it is clear that a more in-depth examinations of negative (eg, frequent use, CUD prevalence, ‘gateway’ relationships with other substance use), or positive consequences (eg, therapeutic benefits for mental health and/or medical conditions, use of safer products and routes of administration), are needed using both quantitative and qualitative approaches.
